# Stepwise engineering of a *Pichia pastoris *D-amino acid oxidase whole cell catalyst

**DOI:** 10.1186/1475-2859-9-24

**Published:** 2010-04-26

**Authors:** Sandra Abad, Jozef Nahalka, Gabriele Bergler, S Alison Arnold, Robert Speight, Ian Fotheringham, Bernd Nidetzky, Anton Glieder

**Affiliations:** 1 Austrian Centre of Industrial Biotechnology, c/o Applied Biocatalysis Research Centre, c/o Institute of Molecular Biotechnology, Graz University of Technology, Petersgasse 14, 8010 Graz, Austria; 2 Institute of Biotechnology and Biochemical Engineering, Graz University of Technology, Petersgasse 12, 8010 Graz, Austria; 3 Institute of Chemistry, Center of Glycomics, Slovak Academy of Sciences, Dúbravska cesta 9, SK-84538 Bratislava, Slovak Republic; 4 Ingenza Ltd., Wallace Building, Roslin BioCentre, Roslin, EH25 9PP, UK

## Abstract

**Background:**

*Trigonopsis variabilis *D-amino acid oxidase (*Tv*DAO) is a well characterized enzyme used for cephalosporin C conversion on industrial scale. However, the demands on the enzyme with respect to activity, operational stability and costs also vary with the field of application. Processes that use the soluble enzyme suffer from fast inactivation of *Tv*DAO while immobilized oxidase preparations raise issues related to expensive carriers and catalyst efficiency. Therefore, oxidase preparations that are more robust and active than those currently available would enable a much broader range of economically viable applications of this enzyme in fine chemical syntheses. A multi-step engineering approach was chosen here to develop a robust and highly active *Pichia pastoris Tv*DAO whole-cell biocatalyst.

**Results:**

As compared to the native *T. variabilis *host, a more than seven-fold enhancement of the intracellular level of oxidase activity was achieved in *P. pastoris *through expression optimization by codon redesign as well as efficient subcellular targeting of the enzyme to peroxisomes. Multi copy integration further doubled expression and the specific activity of the whole cell catalyst. From a multicopy production strain, about 1.3 × 10^3 ^U/g wet cell weight (wcw) were derived by standard induction conditions feeding pure methanol. A fed-batch cultivation protocol using a mixture of methanol and glycerol in the induction phase attenuated the apparent toxicity of the recombinant oxidase to yield final biomass concentrations in the bioreactor of ≥ 200 g/L compared to only 117 g/L using the standard methanol feed. Permeabilization of *P. pastoris *using 10% isopropanol yielded a whole-cell enzyme preparation that showed 49% of the total available intracellular oxidase activity and was notably stabilized (by three times compared to a widely used *Tv*DAO expressing *Escherichia coli *strain) under conditions of D-methionine conversion using vigorous aeration.

**Conclusions:**

Stepwise optimization using a multi-level engineering approach has delivered a new *P. pastoris *whole cell *Tv*DAO biocatalyst showing substantially enhanced specific activity and stability under operational conditions as compared to previously reported preparations of the enzyme. The production of the oxidase through fed-batch bioreactor culture and subsequent cell permeabilization is high-yielding and efficient. Therefore this *P. pastoris *catalyst has been evaluated for industrial purposes.

## Background

**Figure 1:**
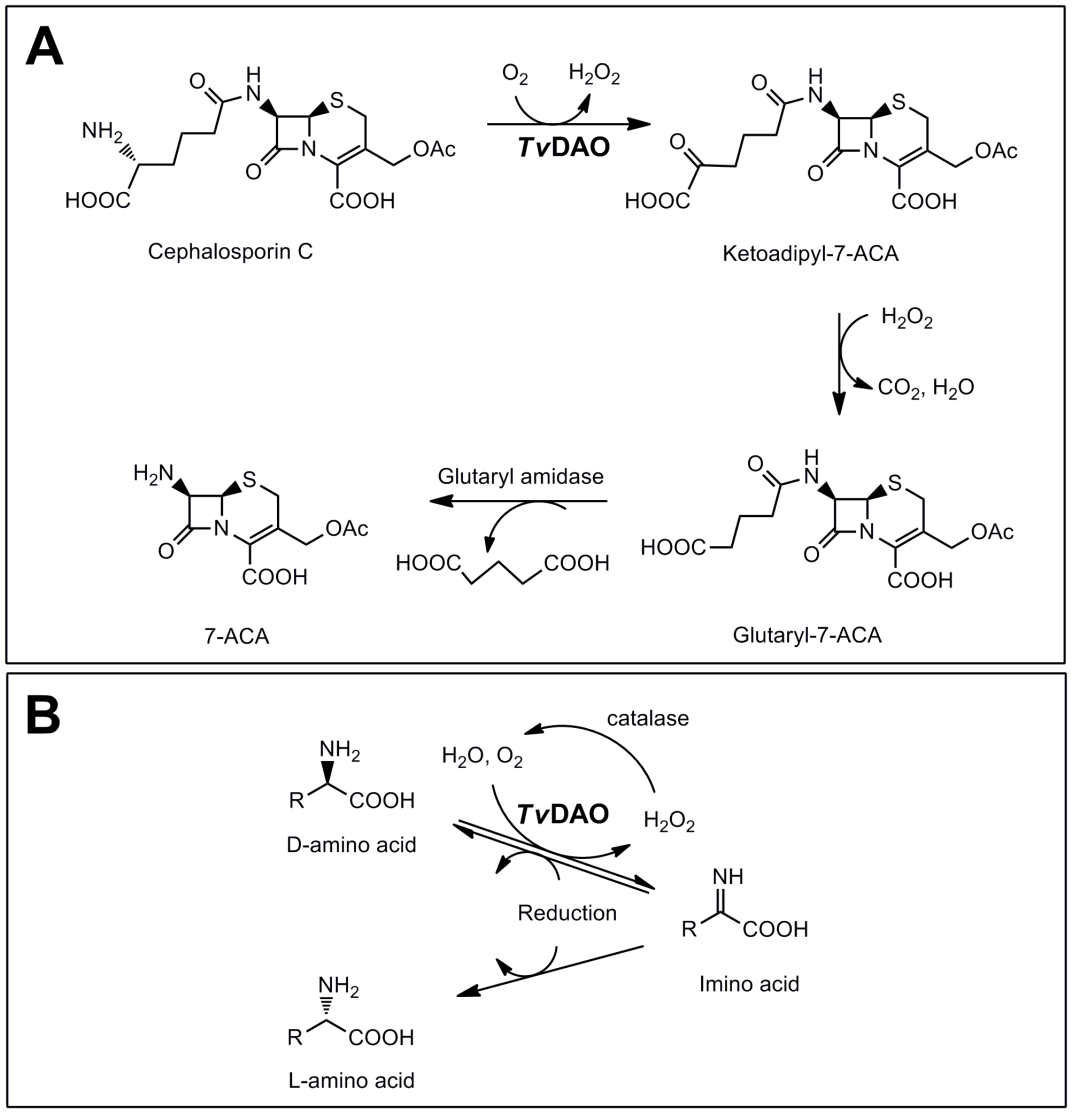
**Industrial applications of *Tv*DAO**. A: Chemo-enzymatic conversion of cephalosporin C to 7-aminocephalosporanic acid. Spontaneous decarboxylation of ketoadipyl-7-ACA (amino cephalosporanic acid) is promoted by the H_2_O_2 _formed in the oxidase reaction of *Tv*DAO. B: Chemoenzymatic production of pure enantiomers of amino acids by amino acid oxidases in combination with imine reduction benefits from the additional presence of a catalase that destroys H_2_O_2_.

D-Amino acid oxidases (DAO, E.C. 1.4.3.3) are well characterized flavoenzymes that have been considered for various applications during the last 20 years. Among these applications, the industrial use of DAO to catalyze the first step of chemo-enzymatic conversion of cephalosporin C to 7-aminocephalosporanic acid [1] is an outstanding example. It represents the only known case of a large-scale (> 1000 tons/year) biocatalytic process employing an oxidase as biocatalyst (Figure 1A). Other promising applications of DAO include the production of α-keto acids [2], analytical determination of D-amino acids in biological samples and foodstuffs [3], the development of DAO biosensors [4] for analytics, and the elimination of traces of D-amino acids from non-natural L-amino acid preparations [5]. More recently, the use of DAO for the chemo-enzymatic synthesis of physiologically active compounds (e.g. L-methionine, phenyl pyruvate, L-6-hydroxynorleucine, L-2-naphtylalanine) has reinforced the applied potential of this enzyme [6]. Figure 1B is a schematic representation of a deracemisation process employing DAO in its first step and scaled up for industrial application [5].

DAO from the yeast *Trigonopsis variabilis *(*Tv*DAO) [7] was the preferred choice of catalyst for cephalosporin C conversion, primarily because it was more efficient on the industrial substrate than other known DAOs, such as enzymes from *Rhodotorula gracilis *and pig kidney for example [8]. *T. variabilis *also showed higher productivity (volumetric and specific) for biosynthesis of DAO than relevant other native producers of the oxidase [8].

The operational stability of *Tv*DAO in biocatalytic conversions has always been a critical issue in process development and has therefore attracted considerable attention. Enzymatic conversion of cephalosporin C depends on O_2 _as co-substrate and furthermore relies on the H_2_O_2 _produced in the enzymatic reaction (Figure 1A). While isolated *Tv*DAO is sensitive to the conditions applied in the process (bubble aeration, high concentration of oxidants), carrier-bound and entrapped immobilisates of the enzyme showed enhanced persistence [9,10]. However, demands on the enzyme with respect to activity and stability but also with respect to the costs incurred vary with the process considered. For simple oxidations in fine chemical production, the H_2_O_2 _usually has to be removed to avoid enzyme inactivation. To our knowledge, a preparation of *Tv*DAO fulfilling the requirements of a broad-scope biocatalyst for fine chemical synthesis was not previously available.

While *Tv*DAO production in the native host presents industrial standard, enhancement of specific as well as volumetric productivity as compared to *T. variabilis *is also desirable (see later). Heterologous expression in *E. coli *has provided oxidase preparations having fine-tuned stability [10,11], activity [12] and even solubility [13]. A number of yeasts including *Kluveromyces lactis *[14], *Saccharomyces cerevisiae *[14], a catalase-deficient strain of *Schizosaccharomyces pombe *[15] and the methylotrophic yeast *P. pastoris *[16,17] have also been employed for recombinant production of *Tv*DAO.

Contrary to cephalosporin C conversion where H_2_O_2 _formed in the reaction of *Tv*DAO is crucial to promote oxidative decarboxylation of the α-keto acid product (Figure 1A), processes for production of chiral amino acids (see Figure 1B) benefit from reduced by-product formation at low peroxide levels (see [18]). A whole-cell biocatalyst producing both *Tv*DAO and catalase in large amounts would therefore be beneficial and the methylotrophic yeast *P. pastoris *seemed to be an ideal point of departure for its development. Heterologous gene expression in *P. pastoris *is often performed under control of the very strong *alcohol oxidase 1 *promoter (P_*AOX1*_) [19] which becomes fully induced when methanol is utilized as the sole carbon source. In addition to promoting recombinant protein production, these conditions also lead to an extensive proliferation of peroxisomes, which can cover up to 80% of the cytoplasmic space [20]. Efficient targeting of recombinant *Tv*DAO into peroxisomes of *P. pastoris *would allow to make efficient use of this intracellular space for the over-expressed protein and at the same time, co-localize the enzyme producing H_2_O_2 _with the one destroying it (i.e. the endogenous catalase [21]). Another plausible advantage of having *Tv*DAO and catalase in the same cellular compartment is that conversion of H_2_O_2 _by catalase recycles O_2 _for the oxidase reaction. Previously described *P. pastoris *strains over-expressing the *Tv*DAO gene displayed only modest productivity [16,17,22]. Driven by a clear industrial demand (Figure 1B) and using a stepwise engineering approach, this paper reports on the successful design and generation of an innovative and notably improved *P. pastoris*-based DAO biocatalyst.

## Results and Discussion

### Expression engineering

Expression engineering to optimise oxidase production involved the re-design of both the gene and the amino acid sequence of *Tv*DAO. The coding gene was optimized for expression in *P. pastoris *according to the codon usage of genes that provide high levels of recombinant protein under methanol induction conditions for P_*AOX1 *_(see Material and methods). Like other DAOs, *Tv*DAO was assumed to be localized in the peroxisomes of the native host [14]. A targeting sequence recognized by either one of the common transport proteins Pex5p and Pex7p is a general requirement for peroxisomal protein import. A bioinformatic analysis of the *Tv*DAO primary structure using TargetP 1.1 [23] and PSORTII [24] revealed the absence of a Pex7p-selective motif PTS2. The occurrence of PTS1, the sequence recognized by Pex5p, was uncertain using publicly available predictor software [25,26] but indicated the C-terminal peptide -Pro-Asn-Leu (PNL) as a putative PTS1. With the aim of enhancing peroxisomal transport of the enzyme under conditions of heterologous expression, we exchanged the native PNL motif by the well known peroxisomal targeting sequence -Ser-Lys-Leu (SKL) which has previously been successfully exploited to achieve peroxisomal localisation of recombinant green-fluorescent protein in *P. pastoris *[27].

Four different gene combinations were obtained by combining native and codon-optimized genes with the C-terminal sequence motifs SKL and PNL. This permutation was necessary because of the currently limited ability to predict the actual success of codon optimization. These DAO variants were combined with the necessary 5'and 3' DNA sequences for expression cassettes delivering chromosomal integration in *P. pastoris *and expression under control of P_*AOX1*_. *Pichia *transformants were cultivated in 96-well format [28] and screened for putative single copy strains using an enzyme-coupled assay for fast detection of DAO activity. This was done under the assumption that expression increases with increasing copy numbers of integrated expression cassettes. Only single copy strains allow the comparison of the effect of individual gene constructs. Quantitative real time-PCR was then used to analyze the selected strains, and the results confirmed that comparable number of expression cassettes (1-2 copies) had been integrated in each strain.

These "low-copy-number" strains were compared with respect to biomass formation and enzyme production in 1.5-liter bioreactor cultivations (Table 1). Among the four strains, the strain Tv1 harbouring the codon-optimized gene for production of *Tv*DAO-SKL showed the highest DAO activity. The exchange of the native putative peroxisomal targeting sequence -PNL to -SKL had no positive effect on enzyme production when the native gene was used (strains Tv5a and Tv1a). However, the same substitution brought a substantial increase in oxidase activity when comparing the codon-optimized genes (strains Tv5 and Tv1). The engineering strategies directed towards codon optimization, which is expected to facilitate translation and optimized subcellular targeting at the same time resulted in a synergistic effect on whole cell *Tv*DAO activity. Strain Tv1 showed an about 5-fold improvement in specific oxidase activity as compared to the reference strain Tv5a. To our knowledge, this is the first report describing a combination of PTS1 exchange with codon optimization of a gene to improve intracellular expression.

**Table 1:** Enzyme and strain engineering for efficient expression of *Tv*DAO in *Pichia pastoris*.

Strain	Sequence		CN	Intracellular activity	Biomass
		
	Codon usage	PTS1		[U/g wcw]	[g wcw/batch]
Tv5a^I^	native	PNL	1	137	220
Tv1a^II^	native	SKL	1	90	274
Tv5^I^	optimized	PNL	1-2	348	259
Tv1^II^	optimized	SKL	1	767	144
Tv1a_mc^II^	optimized	SKL	5	550	240
Tv1_mc^II^	optimized	SKL	16-17	1283	117

^I^PNL sequence: 5'-CCA-AAC-CTT-3'; ^II^SKL sequence: 5'-TCC-AAG-CTG-3'

Enzyme activities are reported for *P. pastoris *cells obtained in 1.5-L bioreactor cultivation employing standard induction with 3 mL/min methanol. Codon usage: native as in the original *Tv*DAO gene, or optimized for expression using the AOX1 promoter. *PTS1 *- peroxisomal targeting using the native C-terminal tripeptide PNL or the engineered motif SKL. *CN*: Number of copies of the expression cassette integrated into the *P. pastoris *genome. wcw: wet cell weight. Activity measurements were performed three times revealing a standard deviation of < 10%.

All low copy strains except Tv1 which integrated a single copy of the expression cassette containing the codon optimized DAO gene with -SKL C-terminal end, allowed biomass concentrations well above 200 g/L using standard *P. pastoris *cultivation procedures including methanol feed in the production phase. A relatively lower biomass yield of 144 g wcw/batch for Tv1 employing the same cultivation conditions might be explained by the stress, which is generated when large amount of recombinant DAO accumulate in the cell. The strain Tv1 grew normally in glycerol containing media where *Tv*DAO expression was repressed, but not during the induction phase using methanol. Similar observations were made with other yeast host organisms, where reduced growth during induction due to an apparent cell toxicity of recombinant DAO was reported [14,16].

### Development of a high-yielding production strain

The codon-optimized gene for *Tv*DAO-SKL was used for further strain development. A new expression construct was made employing the expression vector pPpB1, which facilitates the generation and detection of *P. pastoris *multi-copy transformants. Using activity-based screening of *P. pastoris *transformants, the best active strain denoted Tv1_mc was selected. Characterization by real time-PCR revealed integration of about 16 - 17 copies of the expression cassette. Table 1 shows that the Tv1_mc strain gave a twofold enhancement of specific oxidase activity as compared to the single copy clone Tv1. This result implies that the number of gene copies did not translate linearly into an increasing titre of active *Tv*DAO. Notwithstanding, Tv1_mc is the most efficient host for *Tv*DAO production that has been reported so far. A comparison with earlier reports shows that this strain reached an expression level up to 1.5 and 1 order of magnitude above the expressions levels reported for *E. coli *and *P. pastoris*, respectively [13,17]. Other yeasts such as *S. cerevisiae *and *K. lactis *showed significant lower *Tv*DAO production [14]. Unfortunately, the biomass yield for the multi copy transformant Tv1_mc was even lower than in case of the strain Tv1. As with Tv1, growth was impaired only during induction with methanol. For comparison reasons we constructed another multi-copy strain of *P. pastoris*, termed Tv1a_mc (native gene with -SKL). This strain produced a lower amount of *Tv*DAO activity (550 U/g wcw) than the strains Tv1 and Tv1_mc containing codon optimized genes and it grew normally in the methanol feeding phase. This finding further supports the notion that the recombinant oxidase, at least when it exceeds a certain level in the cell, seems to be inhibitory to *P. pastoris *growth. We therefore considered optimization of the cultivation and induction conditions for recombinant DAO protein production by the best strain Tv1_mc.

**Figure 2:**
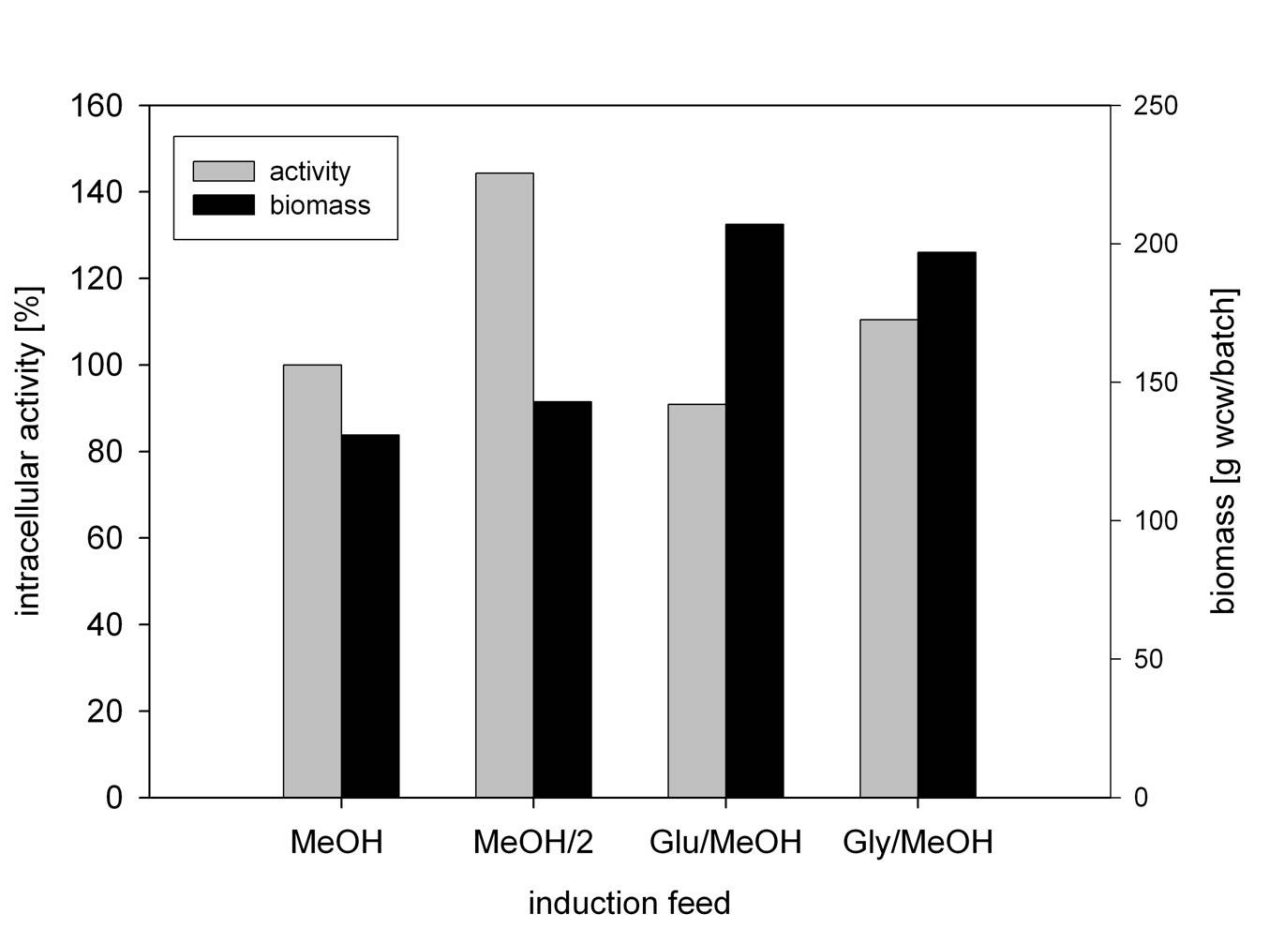
**High-cell-density bioreactor cultivation of *P. pastoris *strain Tv1_mc using induction with a mix-feed strategy**. Values are referenced to oxidase activity obtained using the standard methanol feed and set as 100%. The fed-batch induction phase lasted 90 h in each case. *MeOH*: 3 mL/h methanol. *MeOH/2*: 1.5 mL/h methanol, *Glu/MeOH*: 3 mL/h glucose-methanol mixture, *Gly/MeOH*: 3 mL/h glycerol-methanol mixture. Activity measurements were performed three times, resulting in standard deviations of < 14%.

Using the methanol feed of 3 mL/h as a benchmark, alternative strategies for fed-batch cultivation of Tv1_mc were evaluated. It was known [29-32] that a mixed substrate feed in the induction phase can support *P. pastoris *growth and productivity for the target protein at the same time. Figure 2 depicts the results obtained using feeds of glucose-methanol and glycerol-methanol (each at 3 mL/h) and a reduced pure methanol feed at 1.5 mL/h. The switch from pure methanol feed to mixed substrate feed caused an about 40% increase in biomass yield (~200 g wcw/batch) without compromising the specific activity of the yeast cells. Glycerol-methanol was superior to glucose-methanol at the evaluated feed rate. On the other hand a reduced flow rate (1.5 mL/h) of the methanol feed resulted in a 1.4-fold enhancement of specific activity as compared to the reference, while the biomass yield remained rather low (~140 g/batch).

However the volumetric productivity under these conditions was the highest. The Tv1_mc cells subjected to induction by a reduced methanol feed gave a volumetric oxidase yield of 218 kU/L and a productivity of 2.0 kU/(h L). In comparison the Tv1_mc strain fed with glycerol-methanol resulted in lower yields of 167 kU/L (1.5 kU/hL), and 152 kU/L (1.4 kU/hL) for glucose-methanol induction. These values clearly exceeded those of other known *Tv*DAO expression strains as summarized in Table 2.

**Table 2:** Comparison of relevant host strains used for production of *Tv*DAO.

Expression host	Activity	Reference
*Trigonopsis variabilis*^I^	4,620 U/L^IV^	
*Pichia pastoris*	7,596 U/L^V^	[16]
*Pichia pastoris*	23,000 U/L ^IV^	[17]
*Pichia pastoris*	12,532 U/L^IV^	[22]
*Pichia pastoris*^II^	218,926 U/L^V^	this study
*Saccharomyces cerevisiae*	~110 U/g dcw^V^	[14]
*Kluveromyces lactis*	~150 U/g dcw^V^	[14]
*Escherichia coli*^III^	12,340 U/L^V^	[13]

^I ^Induced with N-carbamoyl-D-alanine

^II ^Induced with reduced methanol feed

^III ^Additional D-methionine induction was reported to yield in a 4.5 fold expression improvement

^IV ^Activity measured with D-alanine as substrate

^V ^Activity measured with D-methionine as substrate

^dcw ^dryed cell weight

Non standardized conditions used for assaying the enzyme activity in different publications make direct and quantitative comparison of published data difficult.

### Whole cell biocatalyst optimization

Due to already described instability issues of the enzyme a cellular "encapsulation" of the oxidase seemed a promising option. Therefore intact cells obtained in a standard bioreactor cultivation of Tv1 (methanol feed of 3 mL/h) were subjected to stability examination. The determination was performed according to a previously reported assay that provides a first-order inactivation constant (*k*_in_) as measure of the operational (in)stability of the oxidase activity [10]. The value of *k*_in _is inversely proportional to stability, according to the relationship τ (half-life time) = ln2/*k*_in_. Preliminary results showed an extraordinary half-life time of more than 10 h for the whole cell catalyst Tv1_mc. However, based on the same oxygen consumption measurements the specific oxidase activity of intact Tv1_mc cells (5 U/g wcw) was less than 1% of the latent intracellular activity, presumably because of severe mass transfer limitations occurring in the intact cell system.

On the other hand the analysis of a lysate preparation after centrifugation was examined as the soluble fraction and the pellet fraction (probably containing partially lysed cells and peroxisomes) and showed the opposite results. The soluble fraction confirmed the high oxidase activity (~1200 U/g wcw) measured before but a high *k*_in _value of 0.0208 min^-1 ^(τ = 0.5 h). Because *k*_in _for the partially lysed pellet fraction was only one-tenth of the value measured for the soluble fraction (*k*_in_^pellet fraction ^= 0.0026 min^-1^; τ = 4.4 h) we still felt strongly encouraged to pursue the development of a whole-cell catalyst. *P. pastoris Tv*DAO-containing Tv1_mc cells served as the starting material. While cell permeabilization was a clear option to attenuate diffusional effects, it was a challenge to prevent a trade-off between enhancement of substrate availability and loss of enzyme stability in the permeabilized whole-cell catalyst. Among various protocols proposed for yeast cell permeabilization [33], the treatment with 2-propanol appeared to be most suitable for our purpose because of its simplicity, efficiency and low costs.

**Figure 3:**
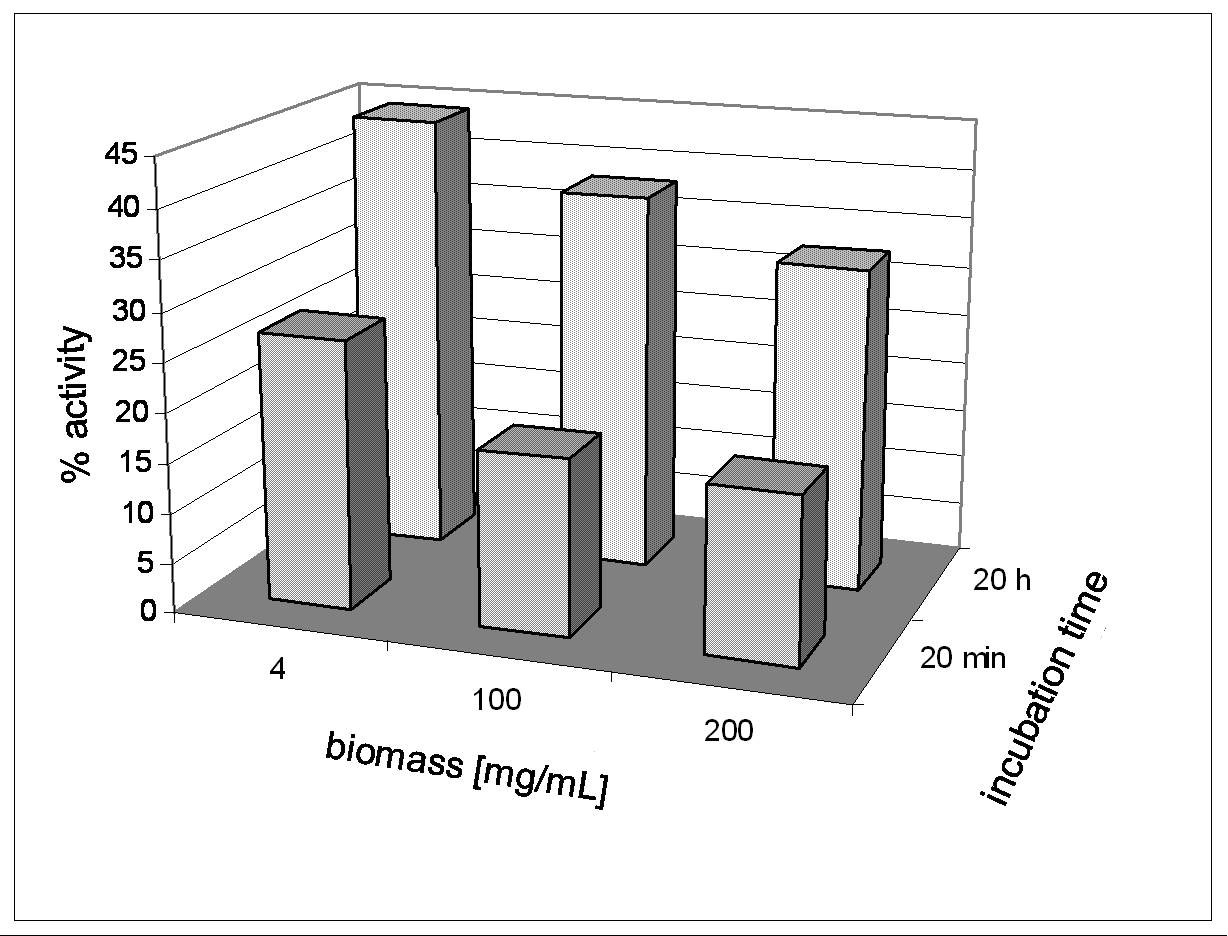
**Effect of biomass concentration on permeabilization of recombinant *Pichia pastoris *cells expressing *Tv*DAO**. Cells of Tv1_mc cultivated in a bioreactor were treated with isopropanol for 20 min and 20 h. The activity of permeabilized cells is given as a percentage of the total intracellular activity (1283 U g/wcw) measured after cell lysis. Activity measurements were performed three times resulting in standard deviations of < 5%.

Batches of Tv1_mc and Tv1a_mc cells (10 g each) containing about 1300 and 500 U/g wcw intracellular activity, respectively, were lyophilized prior to treatment with isopropanol considering that handling of dried biomass is easier and more reproducible than that of wet biomass. Key variables of the permeabilization (concentrations of isopropanol and biomass, incubation time) were examined systematically. Oxidase activity measured in the treated cells was related to the total intracellular activity (Table 3). Freeze-dried cells suspended in buffer were poorly active. However, their incubation in aqueous suspension caused the gradual "appearance" of enzyme activity, reaching ~20% of the maximum available activity after 20 min. Use of isopropanol further enhanced the apparent activity, yielding an effectiveness factor η (= apparent activity/total intracellular activity) of 0.48 (= 627/1300) under optimized conditions where 10% (by volume) of co-solvent was applied. A suitable incubation time was between 5.3 and 10.7 h. Concentrations of isopropanol greater than 10% caused a decrease in η, and incubation times longer than 5 h did not result in further improvement. Selected whole-cell preparations showing high η (> 0.40; Table 3) were analyzed for the stability of the oxidase activity under operational conditions. Values of *k*_in _(hence, the stabilities) were not affected by the permeabilization as compared to the corresponding *k*_in _(*k*_in_^perm. cells ^= 0.0023 min^-1^) reaching at least the stability of the Tv1_mc pellet fraction. Different biomass concentrations in the range 4 - 200 g/L were examined for permeabilization where the highest biomass level reflects the end concentration for standard fed-batch enzyme production in the bioreactor. The value of η obtained through permeabilization in the presence of 10% isopropanol was independent of the biomass concentration although the time required to achieve a maximum level of apparent activity increased with increasing biomass concentration (Figure 3). The stability of the whole-cell catalysts was not affected by the variation of biomass concentration during permeabilization. These results led to the conclusion that the permeabilization of Tv1_mc delivered a novel and promising whole-cell catalyst that was equally active (~500 U/g wcw) but three times more stable than the reported *E. coli *oxidase preparations [13]. The stability of *Tv*DAO in permeabilized lyophilized *P. pastoris *cells relative to the cell free *P. pastoris *preparation was enhanced by one order of magnitude.

**Table 3:** Systematic optimization of process variables for the permeabilization of *P. pastoris *cells expressing *Tv*DAO.

		Accessible activity after permeabilization			
Isopropanol concentration	Incubation time	Tv1a_mc		Tv1_mc	
		
[%, by vol.]	[h]	[U/g wcw]	η	[U/g wcw]	η
-	-	6	~0.01	5	<0.01
0	0.3	100	0.20	321	0.23
4	0.3	118	0.23		
8	0.3	141	0.28		
10	0.0	100	0.20		
10	0.3	184	0.36	430	0.33
10	0.7	193	0.38		
10	1.5	206	0.41		
10	2.5			579	0.45
10	3.0	226	0.45		
10	5.3			627	0.49
10	10.7			621	0.48
12	0.3	176	0.35	424	0.33
15	0.3	170	0.34		
20	0.3	64	0.13		
40	0.3	14	0.03		

The accessible activity is the activity of the whole-cell system after permeabilization, as measured in U/g wcw and expressed as η, the effectiveness factor (= apparent activity/total intracellular activity), whereas the total intracellular activity was determined after cell disruption with a French Press. Strains Tv1a_mc and Tv1_mc contained 550 and 1283 U/g wcw of total intracellular activity, respectively. Lyophilized *Pichia *cells were used for permeabilization with a concentration being constant at 4 mg/mL. Results in the first row referred to experiments carried out with intact cells obtained from one standard bioreactor cultivation. Permeabilization was performed three times per defined condition, with a standard deviation of the effectiveness factors of < 5%.

## Conclusions

A multi-level engineering approach was successfully applied to develop an innovative whole-cell enzyme preparation of *Tv*DAO that is suitable for applications in industrial deracemization processes (Figure 1B). The outstanding capabilities of *P. pastoris *in recombinant protein production were harnessed to provide high specific activity (1.3 kU/g wcw) and, due to high cell density cultivation of *P. pastoris*, also very high volumetric oxidase activities (150 kU/L) under standard induction conditions, which could be improved by a reduced methanol feed (218 kU/L). These values are well above the highest levels reported in literature (see Table 2). Mild permeabilization of the *Pichia *biomass allowed about half of the latent intracellular activity to be utilized, while at the same time retaining the good operational stability of the enzyme entrapped in the cell matrix of *P. pastoris *peroxisomes. Peroxisomal targeting might be a generally useful strategy of producing recombinant oxidases in *P. pastoris*, especially under circumstances where a whole-cell catalyst preparation is considered and immediate removal of the H_2_O_2 _generated in the enzymatic reaction is desired. In addition, when protein expression is induced by methanol induction, increased peroxisome formation provides increased capacity for the expressed and targeted oxidase. Due to these advantages a *P. pastoris*-based whole-cell *Tv*DAO preparation and deracemisation processes (Figure 1B) are being scaled up and the new *Tv*DAO whole cell catalyst can be rapidly integrated into the existing process.

## Methods

### Chemicals and media

Oligonucleotides were purchased from IDT Integrated DNA Technologies BVBA (Leuven, Belgium) or Invitrogen Corp. (Carlsbad, CA, USA). Sterile water was from Fresenius Kabi Austria (Graz, Austria). All DNA-modifying enzymes were obtained from Fermentas GmbH (Burlington, Ontario, Canada). Unless otherwise stated, all chemicals were from Carl Roth GmbH (Karlsruhe, Germany), Becton, Dickinson and Company (Franklin Lakes, NJ, USA) or Sigma-Aldrich (St Louis, MO, USA).

Complex media contained 10 g/L yeast extract, 20 g/L peptone and 20 g/L glucose (YPD). Media for plates were solidified by addition of agar to 1.5% w/v. The minimal media used in this work contained 200 mM potassium phosphate buffer (pH 6.0), 13.4 g/L yeast nitrogen base and 0.0004 g/L D-biotin. They differed with respect to the carbon source concentration: 10 g/L glucose for BMD, 1 and 5% (by vol.) methanol for BMM2 and BMM10, respectively.

### Cloning and engineering of the *Tv*DAO gene, and construction of *P. pastoris *expression cassettes

*Escherichia coli *TOP10F' (Invitrogen Corp.) was used as host for all cloning steps. The protein sequence [GenBank: AAR98816] of D-amino acid oxidase from *Trigonopsis variabilis *ATCC 10679 was employed for the design of a codon optimized gene for over-expression in *Pichia pastoris *under conditions of methanol induction, using the program Gene Designer (DNA2.0, Menlo Park, CA, USA) with a threshold set at 10%, preferring the best codons. Repetitive codons were manually corrected. The optimized codon usage (see Table 4) was calculated from three highly transcribed genes encoding enzymes of the *Pichia pastoris *methanol utilization pathway (alcohol oxidase 1 Aox1 [GenBank: U96967]; dihydroxyacetone synthase Das1 [GenBank: FJ754551]; formaldehyde dehydrogenase Fld [Genbank: XP_002493270]) and from a plant gene (*Hevea brasiliensis *hydroxynitrile lyase *Hb*HNL [Genbank: U40402]) that is efficiently expressed in *P. pastoris *[34]. The resulting gene was made by gene synthesis. The native gene for *Tv*DAO was kindly provided by Ingenza Ltd. (Roslin, UK). Exchange of the putative peroxisomal targeting sequence at the C-terminus of *Tv*DAO was carried out using a PCR employing a suitable modified reverse primer (see Table 1). Phusion™ High-Fidelity DNA polymerase was used in this and all other PCR experiments applying a protocol provided by the supplier (Finnzymes Oy, Espoo, Finland).

**Table 4:** Codon usage table designed for high level expression during methanol induction in *P. pastoris*.

Amino acid	Codon	Frequency [%]	Amino acid	Codon	Frequency [%]
A	GCU	54.3	P	CCU	42.3
	GCC	23.3		CCC	6.0
	GCA	20.5		CCA	52.0
	GCG	2.0		CCG	0.0
C	UGU	70	Q	CAA	66.5
	UGC	30.0		CAG	33.5
D	GAU	33.5	R	CGU	20.2
	GAC	66.5		CGC	0.0
E	GAA	47.0		CGA	1.2
	GAG	53.0		CGG	1.2
F	UUU	31.0		AGA	71.5
	UUC	69.0		AGG	6.0
G	GGU	64.3	S	UCU	48.2
	GGC	8.5		UCC	27.2
	GGA	25.3		UCA	11.3
	GGG	2.3		UCG	4.3
H	CAU	20.0		AGU	4.3
	CAC	80.0		AGC	4.3
I	AUU	52.0	T	ACU	46.5
	AUC	43.7		ACC	38.0
	AUA	4.3		ACA	9.5
K	AAA	25.5		ACG	6.0
	AAG	74.5	V	GUU	48.5
L	UUA	11.5		GUC	29.8
	UUG	43.7		GUA	6.5
	CUU	21.2		GUG	15.3
	CUC	7.7	W	UGG	100
	CUA	2.5	Y	UAU	22.0
	CUG	13.5		UAC	78.0
M	AUG	100	Stop	UAA	75.0
N	AAU	21.5		UAG	0.0
	AAC	78.5		UGA	25.0

### Construction of *P. pastoris *expression cassettes

Products obtained from PCRs were purified with the QIAquick PCR Purification Kit (Qiagen, Hilden, Germany), digested with *Spe*I and *Asc*I (native *Tv*DAO genes) or *EcoR*I and *Not*I (synthetic *Tv*DAO genes). The digested DNA fragments were ligated via *Spe*I/*Asc*I or *Eco*RI/*Not*I restriction sites into equally digested in-house *E. coli *- *P. pastoris *shuttle vectors pPpT2 or pPpB1 (TU Graz strain collection BT 5713 and 5709, respectively) that provide single or multi-copy chromosomal integration of target genes in *P. pastoris*, respectively. Relevant features and sequence elements of these shuttle vectors include: origin of replication of the *E. coli *plasmid pBR322 [35]; the *AOX1 *promoter (P_*AOX1*_) starting with a *Bgl*II site; a multiple cloning site with unique restriction sites for *Eco*RI, *Spe*I, *Asc*I and *Not*I; the *AOX1 *transcription termination sequence; and an antibiotic resistance cassette consisting of a synthetic bacterial promoter called EM72 in tandem with a truncated version of the *P. pastoris ILV5 *(*acetohydroxyacid reductoisomerase*) promoter, a synthetic gene coding for the amino acid sequence of bleomycin (ble) conferring resistance against Zeocin from *Streptoalloteichus hindustanus*. The *ble *gene had been codon optimized for function in *E. coli *as well as *P. pastoris*. The pPpT2 shuttle vector further contained the *P. pastoris AOD *(*alternative oxidase*) transcription termination sequence [36] at the 3' of the coding sequence of the synthetic *ble *gene. The pPpB1 vector had the same additional features except that the *Saccharomyces cerevisiae *ADH1 (alcohol dehydrogenase 1) promoter and terminator controlled the transcription of the *ble *gene.

### Transformation of *P. pastoris*

A Mut^S ^strain derived from *P. pastoris *CBS 7435 (TU Graz strain collection number BT 3132) was used for transformation. A condensed protocol [37] was used in which the shuttle vectors were employed after linearization with *Bgl*II and purification with the QIAquick PCR Purification Kit (Qiagen). Transformations were performed in ice-cold electro-transformation cuvettes (0.2 cm, Cell Projects Ltd., Kent, UK) using pulse at 200 Ù, 25 μF and 1.5 kV. 0.5 mL of ice-cold sorbitol (1 M; in water) was added immediately after the electro shock, and the suspension was transferred to a sterile 12 mL polypropylene tube (Greiner, Frickenhausen, Germany). YPD medium (0.5 mL) was added to the tube which was then incubated for 2 h at 30°C using agitation at 60 rpm. After this regeneration step, aliquots (0.2 mL) were plated on solid selection medium containing 100 mg/L Zeocin.

### Copy number determination by quantitative PCR

The number of copies of *Tv*DAO expression cassettes integrated into the *P. pastoris *genome was determined by quantitative real-time PCR as described in [38] employing the endogenous ARG4 gene as a reference. Power SYBR Green PCR Master Mix (Applied Biosystems, Foster City, CA, USA) was used in an ABI PRISM 7300 Real Time PCR System (Applied Biosystems). The following oligonucleotide primers were applied in a concentration of 250 nM when using 2 ng genomic DNA of *P. pastoris *as template: AOX1-fw-RT (gaagctgccctgtcttaaacctt)/AOX1-rv-RT (caaaagcttgtcaattggaacca) and ARG4-RT-fw (tcctccggtggcagttctt)/ARG4-RT-rv (tccattgactcccgttttgag). The temperature conditions were: 10 min at 95°C; 40 cycles for 15 s at 95°C and 60 s at 60°C followed by a dissociation step (15 s at 95°C, 30 s at 60°C, 15 s at 95°C) at the end of the last cycle.

### Small-scale cultivation of *P. pastoris*

*P. pastoris *transformants were first cultivated in deep well plates (96-well format) using a modified procedure after Weis et al. [28] in a Multitron II stackable incubation system (Infors, Bottmingen, Switzerland). Briefly, following a 60 h long incubation in 250 μL BMD medium (320 rpm, 28°C, 80% air humidity), the cultures were induced by adding 250 μL BMM2. 50 μL BMM10 were additionally supplied after ~70 h as well as after ~84 h total cultivation time. After a 48 h-long induction phase (108 h of cultivation). 50 μL of each culture were transferred to V-bottom microtiter plates (Greiner Bio-One #651101) from which glycerol stocks were prepared. The remainder of the cell suspension was centrifuged (Eppendorf centrifuge 5810R: 3220 × g, 4°C, 10 min) and the pellets were collected for activity determinations. 300 μL Yeast Buster reagent (Novagen, Darmstadt, Germany) were added to the cell pellet which was resuspended and incubated for ~30 min at room temperature using agitation at 1400 rpm using a TITRAMAX 1000 shaker (Heidolph Instruments GmbH & Co. KG, Germany). Following removal of cell debris by centrifugation (Eppendorf centrifuge 5810R: 3220 × g, 4°C, 10 min), 10 μL of the supernatant were employed for measuring *Tv*DAO activity using the photometric assay as described below.

### Bioreactor cultivation of *P. pastoris*

The inoculum (optical density OD_600 _10 - 15) was prepared in two preculture steps in 250 mL baffled shake flasks using 50 mL BMGY (10 g/L yeast extract, 20 g/L peptone, 100 mM potassium phosphate buffer (pH 6.0), 13.4 g/L yeast nitrogen base and 10 g/L glycerol). Incubations were done for about 12 and 8 h at 28°C using agitation at 120 rpm (Certomat BS-1). A 1.5-L fed batch-pro^® ^bioreactor system (DASGIP AG, Juelich, Germany) equipped with a six-bladed Rushton turbine impeller and suitable controllers for pH and dissolved O_2 _was used for biomass and enzyme production. The basal medium of Invitrogen Corp. was modified according to Hellwig et al. [39] and contained 40 g/L glycerol, 0.17 g/L calcium sulphate dihydrate, 2.32 g/L magnesium sulphate heptahydrate, 2.86 g/L potassium sulphate, 7.18 g/L aqueous phosphoric acid (85%), 0.64 g/L potassium hydroxide, 0.22 g/L sodium chloride, 0.6 g/L EDTA disodium dihydrate, 0.20 mL/L antifoam (ACEPOL 83 E; Lubrizol Additives, Wickliffe, Ohio, USA). PTM_1 _trace element solution was added (4.35 mL/L medium) after in situ sterilisation of the basal medium. Its composition was as suggested by Invitrogen, namely 0.2 g/L biotin, 6 g/L CuSO_4 _× 5 H_2_O, 80 mg/L NaI, 3.067 g/L MnSO_4 _× H_2_O, 0.2 g/L Na_2_MoO_4 _× 2 H_2_O, 20 mg/L H_3_BO_3_, 0.916 g/L CoCl_2 _× 6 H_2_O, 20 g/L ZnCl_2_, 65 g/L FeSO_4 _× 7 H_2_O, and 5 mL/L H_2_SO_4_. The pH of the final medium was adjusted to 6.0 using an ammonia solution (25% by volume; technical quality).

The cultivation started from an initial liquid volume of 0.65 L (in total) with 50 mL of the 2^nd ^preculture in a batch phase (28°C, aeration at 0.7 L/min) in which glycerol served as the sole source of carbon. The stirrer speed varied between 500 and 1200 rpm as required to maintain a level of dissolved O_2 _greater or equal 30% air saturation. The ammonia solution was used for pH control and likewise as nitrogen source. Following depletion of glycerol in the batch phase, typically after ~16 - 18 h, glycerol was fed from a 700 g/L substrate solution containing 12 mL/L PTM_1 _solution. The feed flow rate increased exponentially over 6 h from 4.77 mL/h to 11.72 mL/h, afterwards gradually decreased to zero within 2 h. Decreasing glycerol supply was accompanied by the start of methanol feeding (supplemented with 12 mL/L PTM_1 _solution). The flow rate was increased linearly to 3 mL/min within the 2 h where the glycerol feed was reduced and remained constant at this high level for another 90 h. Feeding of mix-substrates (methanol/glucose and methanol/glycerol) occurred in the same manner. Both mix fed contained 452 g/L methanol and 346 g/L glucose or 321 g/L glycerol, respectively and were supplemented with 12 mL/L PTM_1 _solution.

Biomass was harvested using an Avanti J-20 XP centrifuge (Beckman Coulter, Krefeld, Germany) employing a JA-10 rotor at 2831 × g (10 min, 4°C). The pellet was washed once with a 100 mM potassium phosphate buffer (pH 6.0) and stored frozen (-20°C) until further use.

For cell lysis the thawed cell mass (~5 g) was suspended in 10 mL Tris buffer (100 mM, pH 7.5) and passed 2 times through an Aminco French press using an FA-030 cell (SLM Instruments, Rochester, NY, USA) at approx. 150 bar. Insoluble material was separated from the supernatant by centrifugation (20 min, 14000 rpm, 4°C) and further washed three times with 10 mL Tris buffer as above. Both the supernatant and the insoluble fraction were used for the measurement of *Tv*DAO activity.

### Assays for oxidase activity and stability

An enzyme-coupled colorimetric assay for oxidase activity was used as described by Alexeeva et al. [40] with slight modifications. Briefly, 10 μL of appropriately diluted Yeast Buster cell lysate were transferred into a microtiter plate well. Assay solution (190 μL) was added and colour development (measured at 510 nm) monitored for 5 min at room temperature. The composition of the assay solution was 100 mM potassium phosphate buffer (pH 7.8), 0.5 mM 2,4,6-tribromo-3-hydroxybenzoic acid, 0.75 mM 4-aminoantipyrine, 10 mM D-methionine, and 0.025 mg/ml horseradish peroxidase (type VIa; Sigma-Aldrich catalogue number P6782).

A direct assay of *Tv*DAO activity used measurement of O_2 _consumption at 30°C. A previously described glass mini-reactor with a working volume of 30 mL was employed [10]. The reactor was equipped with a fibre-optic oxygen micro-optode (PreSens GmbH, Regensburg, Germany), a temperature sensor, and a teflon sparging tube (1 mm internal diameter) through which air O_2 _was supplied. Mixing was achieved with a magnetic stirrer operated at 300 rpm.

Initial rates of O_2 _conversion were recorded using 10 mM D-methionine as the substrate dissolved in 10 mM Tris buffer (pH 7.5). Reactions were started by adding 20 μL of appropriately diluted *P. pastoris *cell extract, obtained through either lysis or French press disruption of biomass, to 30 mL of reaction mixture. Alternatively, a suitable amount of untreated or permeabilized yeast cells was added. Note the standard protocol for cell lysis which involved mixing of 100 mg wet biomass with 400 μL Yeast Buster reagent followed by incubation of the suspension for 10 min at 4°C. One unit of oxidase activity refers to 1 μmol O_2 _consumed/min under the conditions used. Standard deviation was derived from three independent activity determinations and calculated according to following equation, where *x *= experimental value and *n *= number of independent experiments:

A previously reported procedure was applied for the determination of "operational" stability of the different oxidase preparations [10]. Air was sparged into the mini-reactor at a flow rate of 30 L/h. The substrate solution contained 100 mM D-methionine dissolved in 100 mM Tris buffer (pH 7.5). The reaction was started by addition of a suitable amount of oxidase (cell extract, whole-cell preparation) such that the level of dissolved O_2 _initially dropped to about 50 μM (~25% air saturation). The time course for [O_2_] was then recorded until the concentration of oxygen returned ~100% air saturation. Data were fitted with an exponential decay function to obtain an estimate for the first-order inactivation constant *k*_in _[10].

### Permeabilization

About 10 g wet *P. pastoris *biomass was suspended in 10 mL distilled water and freeze-dried over night using a Christ Alpha 1-4 LSC freeze dryer (Martin Christ Gefriertrocknungsanlagen, Osterode, Germany) operated at 0°C and 0.570 mbar. Permeabilization by isopropanol was carried out in a total volume of 1 mL, using 4 mg dried cells unless otherwise stated. The alcohol concentration was varied in the range 2 - 40% (by volume, in water). Pure water served as reference. Incubation of the cells was done at 4°C without agitation; the time was variable between 20 min and 50 h as indicated. 100 μL cell suspension was used to measure activity and stability.

## Competing interests

The authors SA, JN, GB, BN and AG declare that they have no competing interests. RS is employed by Ingenza Ltd. who is interested in the commercialization of an industrial chemo-enzymatic process for amino acid deracemization.

## Authors' contributions

SA and GB constructed the various *Tv*DAO expression cassettes and carried out *Pichia pastoris *strain construction. They performed the screening work and did the bioreactor cultivations. The screening assay was provided by Ingenza. SA also supervised the RT-PCR experiments and interpreted data concerning protein expression. JN performed cell permeabilization experiments and determined the activities and stabilities of whole-cell catalysts. AA and RS provided feedback about the reproducibility of the lab results in scale up experiments at Ingenza. IF, RS, BN and AG made substantial contributions to the conception, design and discussion of the overall project strategy and the experiments. SA, AG and BN wrote the paper. All authors have read and approved the final version of the manuscript.

## References

[B1] Riethorst W Reichert A An industrial view on enzymes for the cleavage of cephalosporin C Chimia 1999 53 600 607

[B2] Tishkov VI Khoronenkova SV D-Amino acid oxidase: Structure, catalytic mechanism, and practical application Biochemistry (Moscow) 2005 70 40 54 15701048

[B3] Pollegioni L Molla G Sacchi S Rosini E Verga R Pilone MS Properties and applications of microbial D-amino acid oxidases: current state and perspectives Applied Microbiology and Biotechnology 2008 78 1 16 10.1007/s00253-007-1282-4 18084756

[B4] Rosini E Molla G Rossetti C Pilone MS Pollegioni L Sacchi S A biosensor for all D-amino acids using evolved D-amino acid oxidase Journal of Biotechnology 2008 135 377 384 10.1016/j.jbiotec.2008.06.001 18588925

[B5] Beard TM Turner NJ Deracemisation and stereoinversion of α-amino acids using D-amino acid oxidase and hydride reducing agents Chemical Communications 2002 7 246 247 10.1039/b107580m 12120387

[B6] Khoronenkova SV Tishkov VI D-amino acid oxidase: physiological role and applications Biochemistry (Moscow) 2008 73 1511 1518 10.1134/S0006297908130105 19216715

[B7] Kubicek-Pranz EM Rohr M D-amino acid oxidase from the yeast *Trigonopsis variabilis* Journal of Applied Biochemistry 1985 7 104 113 2865242

[B8] Pollegioni L Caldinelli L Molla G Sacchi S Pilone MS Catalytic properties of D-amino acid oxidase in cephalosporin C bioconversion: a comparison between proteins from different sources Biotechnology Progress 2004 20 467 473 10.1021/bp034206q 15058991

[B9] Dib I Nidetzky B The stabilizing effects of immobilization in D-amino acid oxidase from *Trigonopsis variabilis* BMC Biotechnology 2008 8 72 10.1186/1472-6750-8-72 PMC2557008 18798979

[B10] Nahalka J Dib I Nidetzky B Encapsulation of *Trigonopsis variabilis *D-amino acid oxidase and fast comparison of the operational stabilities of free and immobilized preparations of the enzyme Biotechnology and Bioengineering 2008 99 251 260 10.1002/bit.21579 17680679

[B11] Nahalka J Nidetzky B Fusion to a pull-down domain: a novel approach of producing *Trigonopsis variabilis *D-amino acid oxidase as insoluble enzyme aggregates Biotechnology and Bioengineering 2007 97 454 461 10.1002/bit.21244 17089401

[B12] Vikartovska-Welwardova A Michalkova E Gemeiner P Welward L Stabilization of D-amino-acid oxidase from *Trigonopsis variabilis *by manganese dioxide Folia Microbiologica 1999 44 380 384 10.1007/BF02903709 10983233

[B13] Dib I Stanzer D Nidetzky B *Trigonopsis variabilis *D-amino acid oxidase: control of protein quality and opportunities for biocatalysis through production in *Escherichia coli* Applied and Environmental Microbiology 2007 73 331 333 10.1128/AEM.01569-06 PMC1797113 17056691

[B14] Gonzalez FJ Montes J Martin F Lopez MC Ferminan E Catalan J Galan MA Dominguez A Molecular cloning of *Tv *DAO1, a gene encoding a D-amino acid oxidase from *Trigonopsis variabilis *and its expression in *Saccharomyces cerevisiae *and *Kluyveromyces lactis* Yeast 1997 13 1399 1408 10.1002/(SICI)1097-0061(199712)13:15<1399::AID-YEA187>3.0.CO;2-7 9434346

[B15] Isoai A Kimura H Reichert A Schorgendorfer K Nikaido K Tohda H Giga-Hama Y Mutoh N Kumagai H Production of D-amino acid oxidase (DAO) of *Trigonopsis variabilis *in *Schizosaccharomyces pombe *and the characterization of biocatalysts prepared with recombinant cells Biotechnology and Bioengineering 2002 80 22 32 10.1002/bit.10345 12209783

[B16] Zheng HB Wang XL Chen J Zhu K Zhao YH Yang YL Yang S Jiang W Expression, purification, and immobilization of His-tagged D-amino acid oxidase of *Trigonopsis variabilis *in *Pichia pastoris* Applied Microbiology and Biotechnology 2006 70 683 689 10.1007/s00253-005-0158-8 16217653

[B17] Yu J Li DY Zhang YJ Yang S Li RB Yuan ZY High expression of *Trigonopsis variabilis *D-amino acid oxidase in *Pichia pastoris* Journal of Molecular Catalysis B: Enzymatic 2002 18 291 297 10.1016/S1381-1177(02)00109-1

[B18] Fernandez-Lafuente R Rodriguez V Guisan JM The coimmobilization of D-amino acid oxidase and catalase enables the quantitative transformation of D-amino acids (D-phenylalanine) into α-keto acids (phenylpyruvic acid) Enzyme and Microbial Technology 1998 23 28 33 10.1016/S0141-0229(98)00028-3

[B19] Cregg JM Madden KR Barringer KJ Thill GP Stillman CA Functional characterization of the two alcohol oxidase genes from the yeast *Pichia pastoris* Molecular and Cellular Biology 1989 9 1316 1323 10.1128/mcb.9.3.1316-1323.1989 PMC362724 2657390

[B20] Gleeson MA Sudbery PE The methylotrophic yeasts Yeast 1988 4 1 15 10.1002/yea.320040102

[B21] Klei IJ van der Yurimoto H Sakai Y Veenhuis M The significance of peroxisomes in methanol metabolism in methylotrophic yeast Biochimica et Biophysica Acta 2006 1763 1453 1462 10.1016/j.bbamcr.2006.07.016 17023065

[B22] Tan Q Song QX Zhang YW Wei DZ Characterization and application of D-amino acid oxidase and catalase within permeabilized *Pichia pastoris *cells in bioconversions Applied Biochemistry and Biotechnology 2007 136 279 289 10.1007/s12010-007-9026-6 17625234

[B23] Emanuelsson O Brunak S von Heijne G Nielsen H Locating proteins in the cell using TargetP, SignalP and related tools Nature Protocols 2007 2 953 971 10.1038/nprot.2007.131 17446895

[B24] Nakai K Horton P PSORT: a program for detecting sorting signals in proteins and predicting their subcellular localization Trends in Biochemical Sciences 1999 24 34 36 10.1016/S0968-0004(98)01336-X 10087920

[B25] Neuberger G Maurer-Stroh S Eisenhaber B Hartig A Eisenhaber F Prediction of peroxisomal targeting signal 1 containing proteins from amino acid sequence Journal of Molecular Biology 2003 328 581 592 10.1016/S0022-2836(03)00319-X 12706718

[B26] Neuberger G Maurer-Stroh S Eisenhaber B Hartig A Eisenhaber F Motif refinement of the peroxisomal targeting signal 1 and evaluation of taxon-specific differences Journal of Molecular Biology 2003 328 567 579 10.1016/S0022-2836(03)00318-8 12706717

[B27] Monosov EZ Wenzel TJ Luers GH Heyman JA Subramani S Labeling of peroxisomes with green fluorescent protein in living *P. pastoris *cells Journal of Histochemistry and Cytochemistry 1996 44 581 589 10.1177/44.6.8666743 8666743

[B28] Weis R Luiten R Skranc W Schwab H Wubbolts M Glieder A Reliable high-throughput screening with *Pichia pastoris *by limiting yeast cell death phenomena FEMS Yeast Research 2004 5 179 189 10.1016/j.femsyr.2004.06.016 15489201

[B29] Tschopp JF Brust PF Cregg JM Stillman CA Gingeras TR Expression of the *lacZ *gene from two methanol-regulated promoters in *Pichia pastoris* Nucleic Acids Research 1987 15 3859 3876 10.1093/nar/15.9.3859 PMC340787 3108861

[B30] Zhang W Hywood Potter KJ Plantz BA Schlegel VL Smith LA Meagher MM *Pichia pastoris *fermentation with mixed-feeds of glycerol and methanol: growth kinetics and production improvement Journal of Industrial Microbiology and Biotechnology 2003 30 210 215 10.1007/s10295-003-0035-3 12687491

[B31] d'Anjou MC Daugulis AJ A rational approach to improving productivity in recombinant *Pichia pastoris *fermentation Biotechnology and Bioengineering 2001 72 1 11 10.1002/1097-0290(20010105)72:1<1::AID-BIT1>3.0.CO;2-T 11084587

[B32] Lin-Cereghino GP Lin-Cereghino J Ilgen C Cregg JM Production of recombinant proteins in fermenter cultures of the yeast *Pichia pastoris* Current Opinion in Biotechnology 2002 13 329 332 10.1016/S0958-1669(02)00330-0 12323354

[B33] Pscheidt B Glieder A Yeast cell factories for fine chemical and API production Microbial Cell Factories 2008 7 25 10.1186/1475-2859-7-25 PMC2628649 18684335

[B34] Hasslacher M Schall M Hayn M Bona R Rumbold K Luckl J Griengl H Kohlwein SD Schwab H High-level intracellular expression of hydroxynitrile lyase from the tropical rubber tree *Hevea brasiliensis *in microbial hosts Protein Expression and Purification 1997 11 61 71 10.1006/prep.1997.0765 9325140

[B35] Sutcliffe JG Complete nucleotide sequence of the *Escherichia coli *plasmid pBR322 Cold Spring Harbour Symposia on Quantitative Biology 1979 43 77 90 10.1101/sqb.1979.043.01.013 383387

[B36] Kern A Hartner FS Freigassner M Spielhofer J Rumpf C Leitner L Frohlich KU Glieder A *Pichia pastoris *'just in time' alternative respiration Microbiology 2007 153 1250 1260 10.1099/mic.0.2006/001404-0 17379734

[B37] Lin-Cereghino J Wong WW Xiong S Giang W Luong LT Vu J Johnson SD Lin-Cereghino GP Condensed protocol for competent cell preparation and transformation of the methylotrophic yeast *Pichia pastoris* Biotechniques 2005 38 44, 46, 48 10.2144/05381BM04 PMC2504082 15679083

[B38] Abad S Kitz K Hormann A Schreiner U Hartner FS Glieder A Real-time PCR-based determination of gene copy numbers in *Pichia pastoris* Biotechnology Journal 2010 5 413 420 10.1002/biot.200900233 20349461

[B39] Hellwig S Emde F Raven NP Henke M Logt P van Der Fischer R Analysis of single-chain antibody production in *Pichia pastoris *using on-line methanol control in fed-batch and mixed-feed fermentations Biotechnology and Bioengineering 2001 74 344 352 10.1002/bit.1125 11410859

[B40] Alexeeva M Enright A Dawson MJ Mahmoudian M Turner NJ Deracemization of α-methylbenzylamine using an enzyme obtained by in vitro evolution Angewandte Chemie International Edition 2002 41 3177 3180 10.1002/1521-3773(20020902)41:17<3177::AID-ANIE3177>3.0.CO;2-P 12207381

